# Efficacy and Safety of Different Drug Regimens for Tuberculosis Preventive Treatment: A Systematic Review and Meta-Analysis

**DOI:** 10.7759/cureus.38182

**Published:** 2023-04-26

**Authors:** Rima Shah, Tejas Khakhkhar, Bhavesh Modi

**Affiliations:** 1 Department of Pharmacology, All India Institute of Medical Sciences, Rajkot, Rajkot, IND; 2 Department of Pharmacology, Gujarat Medical and Education Research Society (GMERS) Medical College, Porbandar, IND; 3 Department of Community and Family Medicine, All India Institute of Medical Sciences, Rajkot, Rajkot, IND

**Keywords:** efficacy and safety of tpt, drug regimens, tpt, tuberculosis preventive therapy, tuberculosis

## Abstract

Tuberculosis prevention treatment (TPT) is crucial to the eradication of tuberculosis (TB). Through a comprehensive review and meta-analysis, we compared the efficacy and safety of different TPT regimens. We searched PubMed, Google Scholar, and medrxiv.org with search terms Tuberculosis Preventive Treatment, TPT, efficacy, safety, and drug regimens for TPT and all RCT, irrespective of age, setting, or co-morbidities, comparing at least one TPT regimen to placebo, no therapy, or other TPT regimens were screened and those reporting either efficacy or safety or both were included. The meta-analysis data were synthesized with Review Manager and the risk ratio (RR) was calculated. Out of 4465 search items, 15 RCTs (randomized-controlled trials) were included. The TB infection rate was 82/6308 patients in the rifamycin plus isoniazid group (HR) as compared to 90/6049 in the isoniazid monotherapy (H) group (RR: 0.89 (95% CI: 0.66, 1.19; p=0.43). A total of 965/6478 vs 1065/6219 adverse drug reactions (ADRs) occurred in HR and H groups respectively (RR: 0.86 (95%CI: 0.80 0.93); P<0.0001). Efficacy analysis of the rifampicin plus pyrazinamide (RZ) vs H showed that the risk ratio of infection rate was not considerably varied (RR: 0.97 (95% CI: 0.47, 2.03); P=0.94). Safety analysis showed in 229/572 patients developed ADRs in rifampicin plus pyrazinamide as compared to 129/600 ADRs in the isoniazid group. (RR: 1.87 (95% CI: 1.44, 2.43)). Safety analysis of only rifamycin (R) vs H group showed 23/718 ADRs in R vs 57/718 ADRs in H group (RR: 0.40 (95% CI: 0.25 0.65); P=0.0002). Rifamycin plus isoniazid (3HP/R) has no edge over other regimens in terms of efficacy but this regimen was found significantly safer as compared to any other regimens used for TPT. Rifampicin plus pyrazinamide (RZ) was found equally efficacious but less safe as compared to other regimens.

## Introduction and background

The End TB Strategy was inspired by the World Health Assembly's global targets for tuberculosis (TB) prevention, treatment, and control that were established in 2014 [1]. By 2035, this aggressive plan aims to achieve “a world free of TB” - zero fatalities, disorders, and suffering attributable to TB [2]. Within the next 20 years, the worldwide incidence of TB must drop from more than 1250 cases per million people to less than 100 cases per million [3]. Overall, TB incidence is higher in lower and middle countries including India which makes them the important target population for designing any strategy for TB elimination [2,3].

Various strategies have been planned and implemented for achieving this target worldwide and tuberculosis prevention treatment (TPT) has been considered a significant tool for this [4]. The concept of TPT is not new. In Alaska, a community-wide TPT application decreased the prevalence of TB by 17% annually between 1957 and 1977 [4]. Since then, several well-structured clinical studies have verified many regimens as a reliable and secure method of avoiding TB disorder [5]. It is generally agreed upon that it is advantageous to prevent people from developing active TB, particularly those who are at a high risk of reactivation [6]. When used properly, the available treatments may lower the incidence of transition from infection to active disorder by 90% [6]. The TPT's implementation is still a problem in many high-burden nations, including India, which are emerging nations. The reasons are non-compliance, large population size, drug stockouts, lack of awareness and training, etc. [7].

It is necessary to rule out active TB before starting TPT. The general public should not be subjected to extensive TB testing since it is not feasible. Most individuals with TB infection never develop active TB conditions [8]. Testing for TB infection with the available technologies in individuals who are healthy and have a low risk of developing the condition would be poor yield and costly to perform [9]. As a result, a satisfactory, evidence-based, and cost-efficient testing method would be more suitable and effective. It should be emphasized that even though TPT will reduce the risk of disease progression in some cases, the risk of repeat exposure and subsequent reinfection will remain high and reduce the effectiveness of environmental controls, and preventive measures unless all administrative measures and regular use of personal respiratory protection equipment (PRPE) and contact tracing are effectively executed [10]. Adopting TB airborne infection control across the healthcare system of India may benefit from the newly adopted F-A-S-T plan (“Finding TB cases actively, separating securely, and treating efficiently”) [11].

Different drug regimens have been tried for their use in TPT. Problems with using these drugs are related to deciding the exact posology, risk of adverse drug events, and its efficacy as a preventive treatment for TB [6,8,11]. The risk of toxicities is associated with age, underlying liver disease, kidney disease, drug-drug interaction, and duration of treatment. Studies have shown the impact of longer-term preventative therapy in several high-incidence TB environments where the risk of reinfection is considerable [12,13]. A study from Chennai has shown equal comparable efficacy, safety, and tolerability of the 6-M and 36-M isoniazid preventative therapy regimens for HIV-positive individuals [14]. The ideal TPT duration in India is yet uncertain (e.g., longer duration regimens to cover reinfection versus shorter adherence-friendly regimens), and it is probably dependent on logistical and other factors like feasibility. Recent studies utilizing 12-dosage weekly isoniazid and rifapentine regimen (3HP) in a nation with a low incidence of the disease revealed high rates of treatment completion (87%) [15]. This therapy has the potential to increase TPT acceptability and adherence on a global scale [16]. However, it is uncertain if 3HP is effective in areas where drug resistance is more prevalent. With all of these factors taken into account, it is still uncertain which TPT regimen will be most successful in India. Therefore, there is a need to establish guidelines for TPT with careful consideration of posology, adverse drug events, drug resistance, treatment duration for children and adults, and other pharmacokinetics and pharmacodynamics parameters.

Therefore, the existing meta-analysis and systematic review are planned to comparatively assess and evaluate different drug regimens used for TPT.

## Review

Methodology

Protocol and Registration

The present systematic review and meta-analysis were done as per the “PRISMA (Preferred Reporting Items for Systematic Reviews and Meta-Analyses)” statement. The protocol has been registered with the “PROSPERO (International Prospective Register of Systematic Reviews)” database; the protocol number is CRD42022358479.

Search strategy and selection criteria: Regardless of age, location, the results of the baseline tuberculin skin test (TST), comorbidities like HIV infection, or other factors, we developed a search method to find all randomized-controlled trials (RCTs) comparing at least one TPT regimen to placebo, no treatment, or other TPT regimens among all populations. Results must have been recorded for at least one of the following: effectiveness (based on the prevalence of TB that has been clinically or microbiologically confirmed to be active) or safety (reporting of any of the adverse events reported during the study period or while follow-up). We searched PubMed, Google Scholar, and medrxiv.org. The search terms were Tuberculosis Preventive Treatment, TPT, efficacy, safety, and drug regimens for TPT.

Inclusion criteria: We have included only RCTs of different drug regimens for TPT coming across using the mentioned search terms in the mentioned search engines with no language restriction for inclusion. The last search was run on 18 July 2022.

Exclusion criteria: We had excluded observational, non-comparative, in-vitro, and animal studies and also the studies where full text could not be accessed were excluded from the analysis.

Data extraction: All the retrieved studies-related data were recorded using a predesigned data collection form by two independent researchers independently. With the help of a third researcher, disagreements were settled by consensus. Information was taken from both trials if they provided additional information, including long-term follow-up in a different document. We extracted the following data in Microsoft Excel 365: author, publication year, location, total participants, demographic data in treatment arms (age, gender), the regime used for preventive treatment, follow-up, incidence rate, and adverse drug reactions (ADRs).

Quality assessment of the studies included: Two authors (RS and TK) independently rated the RCTs' methodological quality according to the Cochrane Collaboration risk of bias 2 tool (ROB-2), classifying each as high, low, or with some complications [17,18] and found to have a low risk of bias.

The assessment of publication bias was done using the funnel plot method.

Outcome measures: Comparing the infection rate between individuals who got a TPT drug regimen was the primary outcome variable. The secondary outcome variable was to compare ADRs during the follow-up period.

Data synthesis: An overall risk ratio (RR) was used for these outcomes. Heterogeneity was assessed using I2 and I2 greater than 50% was taken as substantial heterogeneity. Due to the lack of or low heterogeneity, the Mantel-Haenszel technique with a fixed-effect model was used to calculate a RR with a 95% CI (confidence interval) (I2=0 to 50). A version of Review Manager (RevMan, The Nordic Cochrane Centre, The Cochrane Collaboration, Copenhagen, 2014) (v. 5.4.1) was used to synthesize the meta-analysis data.

Results

**Figure 1:**
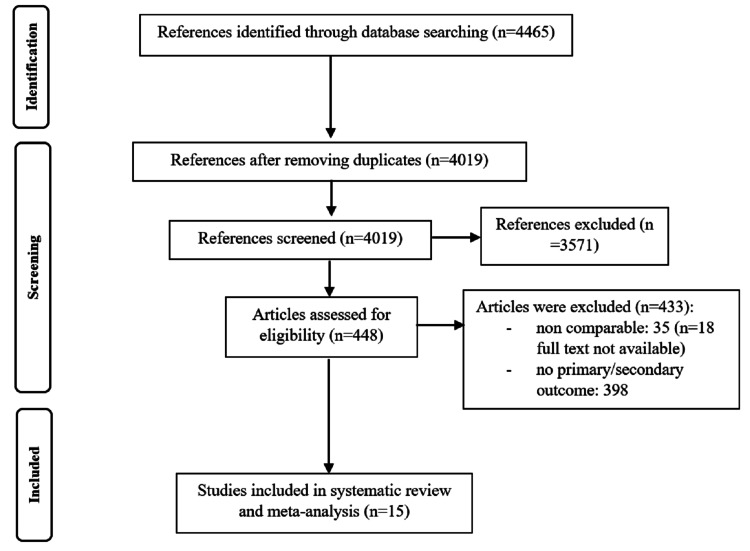
PRISMA flow chart PRISMA: Preferred Reporting Items for Systematic Reviews and Meta-Analyses

Out of 4465 search items, a total of 15 RCTs (15-30) were considered in this meta-analysis and systematic review as per PRISMA protocol (Figure 1).

Table 1 represented the detailed variables of the studies that were included viz year, reference, location, study design, total respondents, age, gender, and regimen used for preventive treatment and follow-up.

**Table 1:** Study characteristics included for the analysis

Reference	Year	Location	Study design	Total Participants	Age (years)	Gender (M: F)	The regime used for preventive treatment	Follow-up
Chan et al. [19]	2012	Taiwan	The randomized, open-label, two-arm, active-controlled trial, parallel-group; stratified by HCV and HBV status	373	> 18 years	Males only	4m daily R vs. 6m H	"Safety outcomes: End of the treatment period in each arm (four months and six months); Efficacy outcome: three years"
HKCS et al. [20]	1992	China	Randomized, double-blind, four-arm, parallel-group, placebo-controlled (double dummy) trial	512	Less than 65 years	Males only	3R, 3Placebo +3H, 3Placebo vs. 6h, 6Placebo	2 to 5 years
Leung et al. [21]	2003	China	Randomized, parallel-group, two-arm, active-controlled, open-label, trial	77	Adults	Mostly men	2m daily RZ vs. 6m daily H	up to five years
Magdorf et al. [22]	1994	Germany	Randomized, three-arm, active-controlled, open-label, parallel-group, trial	150	Children less than 18 years old	Both genders	4m daily R, 2m daily RZ vs. 6m daily H	2 years
Martinson et al. [23]	2011	South Africa	Randomized open-label study	1148	Median 30.4 (IQR, 26.4-34.7)	Both genders	6H, 3HR, 3HP, 72H	(median): 6H: 46.8; 3HP: 48; 72H: 46.8
Menzies et al. [24]	2004	Canada	Randomized, parallel-group, two-arm, active-controlled, open-label, trial	116	18 years and above	Both genders	4m daily R vs. 9m daily H	4 to 9 months
Menzies et al. [25]	2008	Brazil, Canada, Saudi Arabia	Randomized, parallel-group, two-arm, active-controlled, open-label, trial	847	18 years and above	Both genders	4m daily R vs. 9m daily H	4 to 9 months
Rivero et al. [26]	2003	Spain	Randomized controlled trial	319	Mean, 32.7	Around 67-70% of males in all groups	6H, 3HR, 2RZ, no treatment	6H: 12.7; 3HR: 14; no therapy: 19.6
Rivero et al. [27]	2007	Spain	Randomized controlled trial	316	Mean 31.3-33	70-80% of males	6H, 3HR, 2RZ	6H: 12.8; 3HR: 12.6
Sanchez-Arcilla et al. [28]	2004	Spain	A randomized, parallel-group, two-arm, active-controlled, open-label trial	172	18 years or older; SD 12.8 years; Mean age 42.3 years	Men 67% (116); Women 33% (56)	2m daily RZ vs. 6m daily H	2 months and 6 months
Sterling et al. [29]	2011	USA, Canada, Brazil, Spain	A randomized, two-arm, multicentre, parallel-group, open-label, active-controlled, phase III, non-inferiority trial	8053	>2 years old	Males or non-pregnant, non-nursing females.	3m weekly H+Rifapentin vs 9m daily H	33 months
Sterling TR et al. [30]	2016	USA	randomized non-inferiority, open-label trial	7731	More than 12 years; Median 35; 36 in two groups (IQR-25-46; 24-47)	53.5% and 55.4% of males in both groups	3HP vs 9H	24 months
Swindells et al. [31]	2019	10 countries in Africa, Asia & America	Randomized open-label study	3000	Median 35 (IQR, 28-43)	54% females	9H, 1HP	9H: 39.1; 1HP: 39.5 (follow-up median 3.3 years)
Tortajada et al. [32]	2005	Spain	Cluster-randomized (by households), parallel-group, multi-center, active-controlled, open-label trial	352	>1 year old; (trial recruited participants old 1 to >35 years)	Both genders	6m daily H vs. 2m daily RZ	Unclear
Whalen et al. [33]	1997	Uganda	Randomized placebo-controlled trails	2736	Adults with HIV; mean age: 30 years	Both gender, 31 and 32% of males in placebo and treatment groups respectively	6H, 3HR, 3HRZ, placebo	2.5 years

Efficacy Analysis of HR/HP vs H

**Figure 2:**
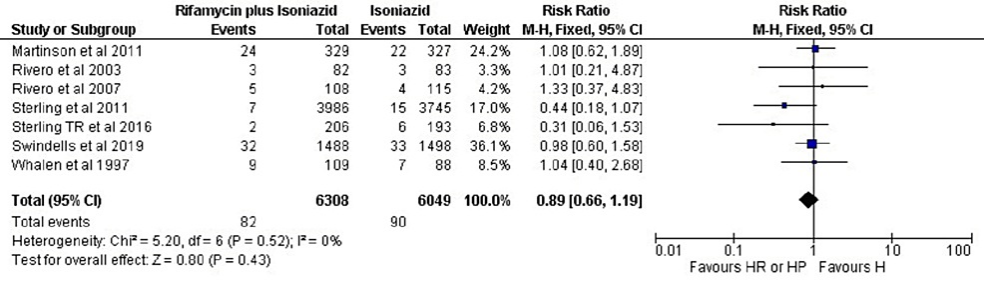
Meta-analytic summaries of infection rate (rifamycin plus isoniazid versus isoniazid) through fixed effect model

As shown in Figure 2, in the rifamycin plus isoniazid group, 82 patients developed TB out of a total of 6308 patients. Whereas, in the isoniazid monotherapy group, 90 patients developed TB out of a total of 6049 patients. This result indicated that the prevalence rate of TB in both study groups did not vary significantly (P=0.43) (RR: 0.89 (95% CI: 0.66, 1.19)). There was no heterogeneity (I2 = 0%) in the outcome.

Safety Analysis of HR/HP vs H

**Figure 3:**
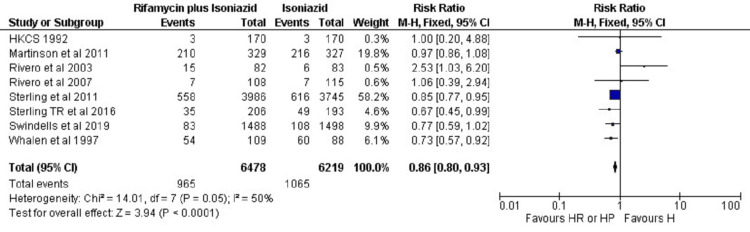
Meta-analytic summary of ADRs (rifamycin plus isoniazid versus isoniazid) through fixed effect model ADRs: adverse drug reactions

As shown in Figure 3, in the rifamycin plus isoniazid group, 965 ADRs occurred in a total of 6478 patients. Whereas, in the isoniazid monotherapy group, 1065 ADRs occurred in a total of 6219 patients. This result indicated that the ADRs of TPT regimes in both study groups were significantly different (P<0.0001) (RR: 0.86 (95% CI: 0.80 0.93)). The heterogeneity was I2 = 50% in this outcome.

Efficacy Analysis of Rifampicin Plus Pyrazinamide versus Isoniazid

**Figure 4:**
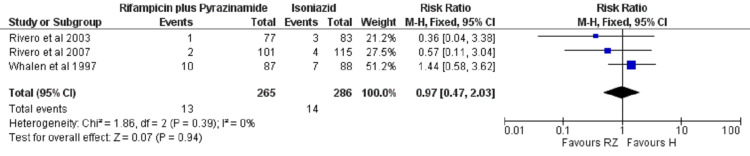
Meta-analytic summary of infection rate (rifampicin plus pyrazinamide versus isoniazid) through fixed effect model

The RR of infection rate was also not significantly different (P = 0.94) in the rifampicin plus pyrazinamide group as compared to isoniazid monotherapy as TPT. Amongst patients treated with rifampicin plus pyrazinamide, 13 infections occurred out of a total of 265 cases while in patients treated with isoniazid, 14 out of 286 cases got infected with TB (RR: 0.97 (95% CI: 0.47, 2.03)). Also, there is no heterogeneity (I2 = 0%) in this outcome (Figure 4).

Safety Analysis of Rifampicin Plus Pyrazinamide versus Isoniazid

**Figure 5:**
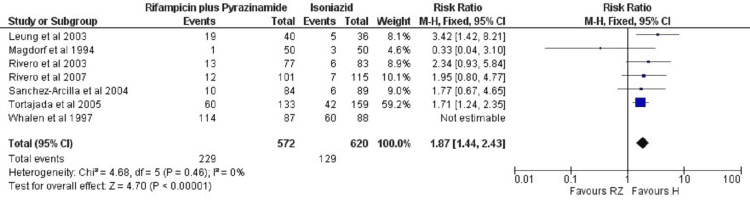
Meta-analytic overviews of ADRs (rifampicin plus pyrazinamide versus isoniazid) through fixed effect model ADRs: adverse drug reactions

The RR of ADRs was significantly different (P < 0.00001) in the rifampicin plus pyrazinamide group as compared to isoniazid monotherapy as TPT. Amongst patients treated with rifampicin plus pyrazinamide, 229 ADRs occurred out of a total of 572 cases while in patients treated with isoniazid, 129 ADRs occurred out of 620 cases (RR: 1.87 (95% CI: 1.44, 2.43)). The heterogeneity (I2 = 0%) was not found in this outcome (Figure 5).

Safety Analysis of R vs H

**Figure 6:**
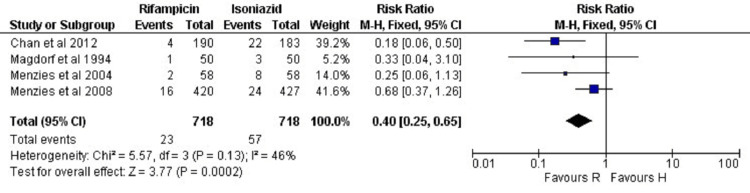
Meta-analytic overviews of ADRs (rifampicin versus isoniazid) through fixed effect model ADRs: adverse drug reactions

As shown in Figure 6, in the rifamycin group, 23 ADRs occurred amongst a total of 718 patients. Whereas, in the isoniazid group, 57 ADRs occurred amongst a total of 718 patients. This finding suggested that there was a considerable variation (P=0.0002) for ADRs of TPT regimes in both the study groups (RR: 0.40 (95% CI: 0.25 0.65)). The heterogeneity was I2 = 46% in this outcome.

Discussion

TPT has been accepted as an effective measure for the prevention of TB worldwide. The adoption of such an intervention has been limited globally despite a wealth of high-quality research demonstrating the effectiveness of preventative treatment for TB and guidelines from the WHO and others [31]. This meta-analysis is attempted to compare and evaluate different TPT regimens in terms of their efficacy and safety so that they can be implemented on large scale in society.

The findings of this study show that combination-drug therapy is better than single-drug therapy for TPT. When the concept of TPT was introduced, the TPT regimen was given to only PLHIV and they were taking isoniazid every day for six to nine months [34]. Due to issues about toxicity and the extensive length of the medication, acceptability, and compliance with daily isoniazid therapy among PLHIV have been low globally [35]. On the basis of randomized studies demonstrating equal effectiveness, improved tolerability, and greater treatment completion compared to daily isoniazid for six months, WHO suggested a three-month (12-dosage) weekly isoniazid plus rifapentine regimen in 2018 [36,13,29]. Any drug should be selected based on its efficacy, safety, suitability (compliance), and cost as per the principles for rational therapeutics [36] but the majority of the studies pertaining to TPT were focused on compliance by the patients only. There is a need to generate studies more focusing on the efficacy and safety of drug dosage regimens for TPT. In Canada, the US, Spain, and Brazil, Sterling contrasted the effectiveness of 3HP to nine months of daily isoniazid monotherapy (9H) in preventing active TB illness. In comparison to 3745 individuals in the 9H arm, 3986 subjects were randomly allocated to the 3HP arm [29]. Adults with HIV were enrolled in a randomized study by Martinson et al. in Soweto, South Africa, to compare the effectiveness of 3HP (328 subjects) to six months of isoniazid monotherapy (328 subjects) [13]. The weekly dosage of 3HP was given to PLHIV in both trials while being carefully monitored by a medical professional (directly observed treatment, or DOT), which helped to maintain adherence and gave scope for monitoring and controlling side effects. If a health professional recorded that a participant had taken at least 90% (11 of the 12 prescribed doses) of their prescribed dosages, the participant was deemed to have finished their 3HP therapy. Using DOT, 3HP treatment completion rates of up to 95% were seen, in comparison to 83.8% for isoniazid monotherapy [13]. Whenever a 3HP regimen is administered, it requires only 12 doses which can improve patient compliance and all other logistic problems as compared to a 6H regimen requiring 182 doses.

The adoption and completion of 3HP among PLHIV and healthcare professionals in low-income nations, however, may be impeded by DOT. These difficulties include the lack of time to visit weekly clinic appointments, which may cost up to 40% of the median weekly income for individuals [37], and the increasing workload for healthcare professionals. SAT (self-administered therapy) helps to alleviate some of such issues, but in other situations, it may also result in a slower detection of side effects and a lower rate of treatment completion [38]. Selecting a shorter but effective drug regimen can also reduce the burden on the health care system. Also, appropriate technology whenever feasible can reduce the workload/burden of the healthcare providers for TPT monitoring and other relevant aspects.

Rifampicin (R), isoniazid (H), pyrazinamide (PZA or Z), and ethambutol (EMB or E) all can be useful as agents for tuberculosis prevention but monotherapy led to a longer duration of treatment as well as increases the chances of the drug resistance when used in large communities. Increasing resistance can be more troublesome for treating active TB cases as we are using the same drugs for it. It is advisable to use combinations of drugs for TB prevention. This metanalysis has compared all available treatment regimens used by different researchers and categorized the regimens into mainly three: HR/HP vs H, RZ v H, and a few studies comparing R vs H. It is identified that the addition of pyrazinamide to the rifampicin does not give any significantly better outcome in terms of infection rate for TB while it increases the incidence of the ADRs. Being bacteriostatic in nature, ethambutol is also not preferred for the TPT regimen.

The primary benefit of adding PZA for TPT is that it eradicates non-replicating persisters that are resistant to conventional TB medications. PZA differs from conventional antibiotics in that it inhibits several targets, including those involved in energy synthesis, trans-translation, and maybe the pantothenate/coenzyme A needed for persister survival [39]. The combination of Z with R is synergistic and helps curtail the duration of the TPT regimen as compared to H alone. But the increased incidence of ADRs has been reported with it and it is significantly higher than in group H alone [39]. The risk-benefit assessment would exclude the use of the RZ combination for TPT.

According to a study, PZA and EMB treatment combined for latent TB infection following exposure to MDR-TB were linked to a significantly high rate of drug-induced hepatitis (50%; occurred after an average of four months of therapy), as well as, to a lesser extent, GI signs that caused 58% of treated contacts to stop receiving treatment [40]. After EMB and PZA were stopped, the levels of SAT and ALAT reverted to normal [40]. High toxicities related to PZA and EMB hamper their use as preventive therapy for TB on large scale in the community.

The WHO currently views rifamycin-containing regimens (3HP and 3HR) as equally recommended for 6H or 9H, irrespective of the HIV status of the subject [41]. Rifamycin-containing regimens are as effective as 6 to 12H in avoiding active TB, may be more successful in averting mortality, and have a reduced risk of liver damage, according to research by Yanes-Lane et al. [42]. However, the possibility of drug-drug reactions, especially with ART, is a significant potential restriction of rifamycin-containing regimens for PLHIV. For instance, a recent trial employing the drugs dolutegravir and rifapentine in healthy volunteers was discontinued due to the high incidence of adverse medication reactions [43]. However, recent research on PLHIV showed that 3HP and dolutegravir may be given together without risk [44]. Dosage modifications are not necessary when using rifamycin and efavirenz together [45]. When recommending rifamycin-containing regimens for PLHIV, it is advisable to examine more recent resources since the pharmacokinetic effects of anti-TB medication on ART (and vice versa) are very variable and often need dosage modifications.

Overall, this review and meta-analysis have confirmed that 3HP has comparable effectiveness to prevent active TB to that of daily INH monotherapy, with fewer adverse events. A significant drawback of this evaluation is how the estimates of TB incidence are affected by the prevalence of TB in the study setting and possible confounding caused by variations in post-treatment follow-up times. Another drawback of the review is that there isn't much research examining TPT regimens in children and pregnant women, which limits the generalization of our findings to such groups. Current recommendations for TPT in children with HIV of all ages include 3HR and isoniazid-based regimens; additional data are still required for 3HP in children under the age of two. As per NTEP India, the 6H regime has been adopted for prophylaxis in children below six years. There is an urgent need for rifamycin-based regimens in pregnant HIV-positive women considering the new signals of significant isoniazid toxicity in such participants. Further data on the additional factors affecting the implementation of 3HP, such as efficacy in children aged two years, drug interactions with ARVs, the impact of self-administration, and cost-effectiveness, are urgently needed. Also, qualitative studies focus on the barriers to implementation of the TPT and measures to overcome that should be planned to meet the goal of ending TB in India.

## Conclusions

Our review concludes that the preventive effect of 3HP/R is similar to that of INH monotherapy or any other treatment regimen for TPT. In addition, 3HP/R was shown to be associated with fewer adverse events as compared to RZ or H regimen. Implementation of this short course of prophylaxis therapy can be easy to implement as the number of doses are less and also reduce the burden and workload of the healthcare system.

## Figures and Tables

**Figure 7:**
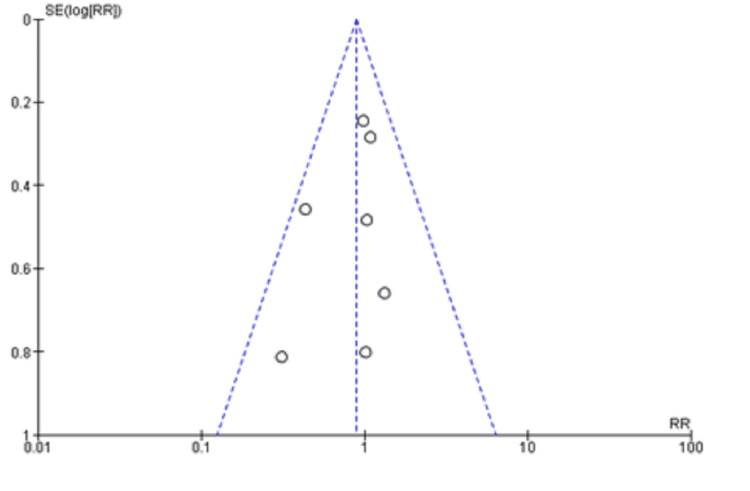
The funnel plot of infection rate (rifamycin plus isoniazid versus isoniazid)

**Figure 8:**
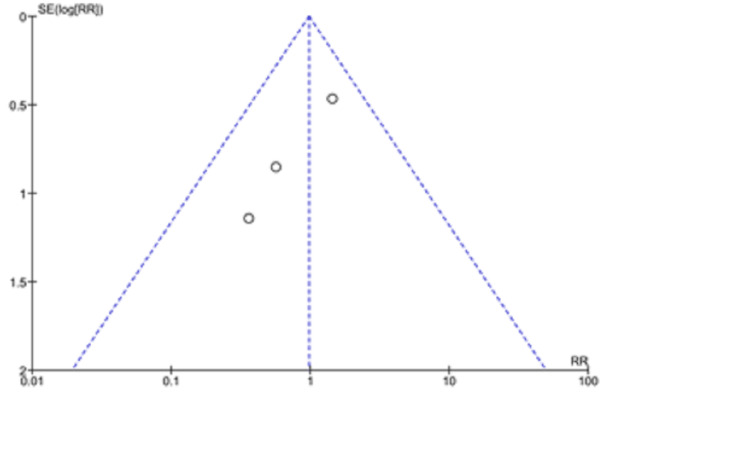
The funnel plot of infection rate (rifampicin plus pyrazinamide versus isoniazid)

**Figure 9:**
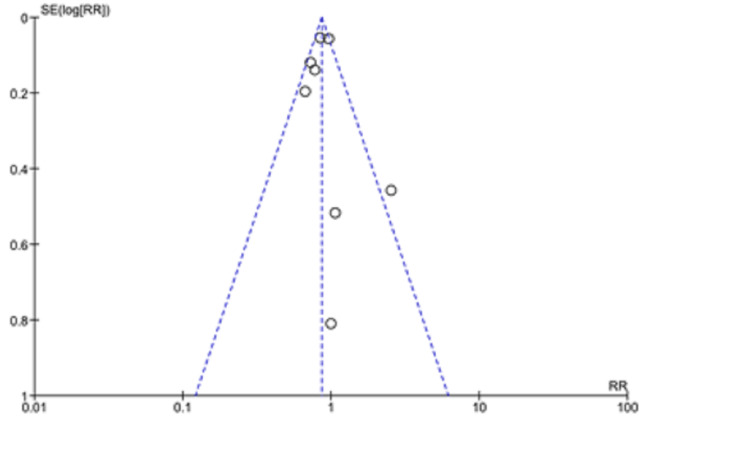
The funnel plot of ADRs (rifamycin plus isoniazid versus isoniazid) ADRs: adverse drug reactions

**Figure 10:**
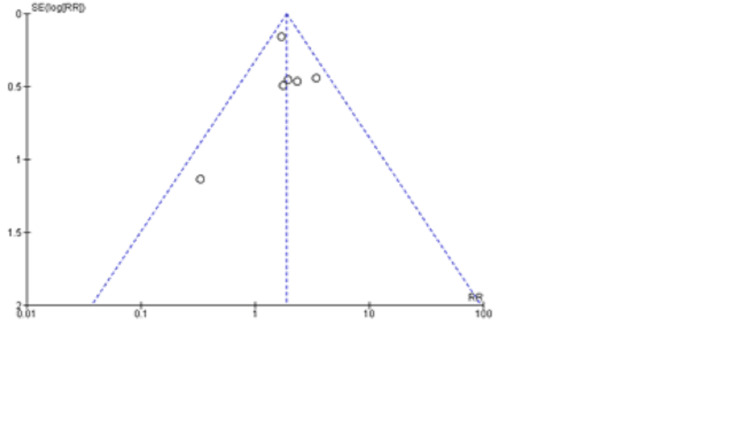
The funnel plot of ADRs (rifampicin plus pyrazinamide versus isoniazid) ADRs: adverse drug reactions

**Figure 11:**
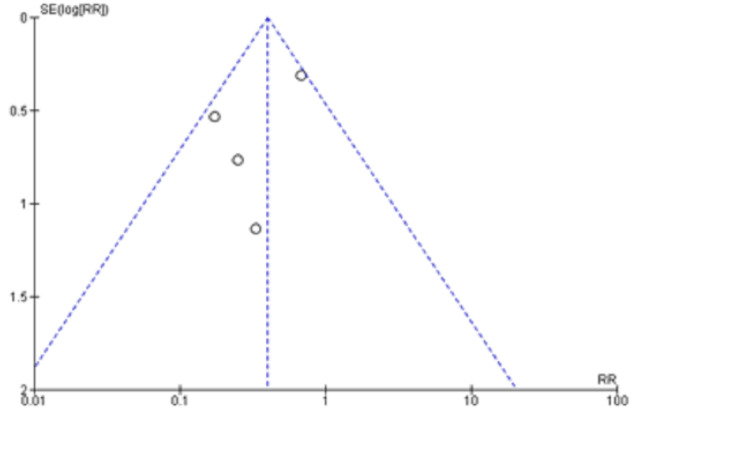
The funnel plot of ADRs (rifampicin versus isoniazid) ADRs: adverse drug reactions

## Appendices

Rifamycin plus isoniazid vs isoniazid as TPT (Figure 7).

Rifampicin plus pyrazinamide vs isoniazid as TPT (Figure 8).

Adverse drug reactions: Rifamycin plus isoniazid vs isoniazid as TPT (Figure 9).

Adverse drug reactions: Rifampicin plus pyrazinamide vs isoniazid as TPT (Figure 10).

Adverse drug reactions: Rifampicin vs isoniazid as TPT (Figure 11).
